# Not only green: Sustainability and debt capital markets

**DOI:** 10.1016/j.jimonfin.2025.103319

**Published:** 2025-04

**Authors:** Annette Becker, Serena Fatica, Michela Rancan

**Affiliations:** a Deutsche Bundesbank, Mainzer Landstraße 46, 60325, Frankfurt am Main, Germany; b European Commission – Joint Research Centre, and MoFiR, via E. Fermi, 21027, Ispra (Varese), Italy; c University of Milan, Via Conservatorio 7, 20122, Milan, Italy

**Keywords:** Sustainable finance, Green bonds, Social bonds, Sustainability bonds

## Abstract

Using a large international sample of corporate borrowers over the period 2014–22, we study the determinants of issuing green, sustainability and social (GSS) bonds. First, we document a remarkable growth of the GSS segment in the most recent years, possibly spurred by the public commitment towards financing a sustainable economic recovery after the COVID-19 pandemic. The results from a multinomial logit for the choice of bond type confirm that countries’ sustainability stance acts as an incentive for corporate access to the sustainable bond segment. Moreover, borrowers in sectors that are green or can become green, as well as those that have already issued and committed to external assurance on the GSS segment, are more likely to raise funds with non-conventional securities.

## Highlights

• The market for non-conventional bonds aimed at financing investments with sustainable purposes has grown exponentially in the recent years.
• Firms in countries with pro-sustainability policies and previous issues of sovereign green, sustainability and social (GSS) bonds are more likely to borrow in the non-conventional bond segment.
• Borrowers engaging in activities that are green or can become green and those that have already issued and committed to external assurance on the GSS segment are more likely to raise funds with non-conventional securities.

## 1 Introduction

As capital markets have been devoting increasing attention to sustainability issues, innovative debt instruments have emerged to finance investment projects that bring environmental, climate and broad sustainability benefits. The market for green, sustainability and social (GSS) debt securities has grown exponentially over the past years, on the back of the sustained demand for green bonds.^1^

Green, sustainability and social bonds are debt securities intended to raise funds for investments that have environmental, climate and social benefits. The emphasis on the use of proceeds (UoP) is a specific feature of GSS bonds, which, otherwise, are contractually and financially similar to conventional fixed income securities. The inception of the sustainable bond market dates back to the 2000s, when the first social bond (2006) and the first green bond (2007) were issued by the International Finance Facility for Immunization (IFFM) and by the European Investment Bank (EIB), respectively. Since then, not only supranational institutions but also corporates and financial institutions have increasingly relied on green securities in their financing choices. The market developed further in 2016 with the first sovereign green issuance by Poland. To date, green bonds are an important instrument in corporate finance as well as in public finance.

More recently, in response to strong demand for sustainable investment products and the need to raise funds to pursue broader sustainability goals, other types of debt instruments, notably sustainability bonds and social bonds, have gained traction. These debt securities differ from green bonds mainly in the type of projects for which the money is raised. Based on the reported UoP, green bonds appear to be predominantly issued to finance investments for climate change mitigation and decarbonization purposes. In a large set of Fitch-rated green securities, renewable energy and energy efficiency together account for half of the rated UoP (Fitch, 2023).^2^ Clean transportation and green buildings are targeted by roughly one third of the reported UoP.^3^ Sustainability bonds present a mix of green and social UoP, with the former being the predominant component. Among Fitch-rated sustainability bonds, green buildings is the most common category, which supports the notion that these instruments are best suited to finance investments where environmental and social benefits are blended. Affordable housing, socioeconomic empowerment and advancement, and affordable basic infrastructure appear among the most commonly reported categories of expenditure that motivate the issuance of social bonds.

Several factors have contributed to the rapid growth of the GSS segment. The introduction of international guidelines has promoted the standardization of market and reporting practices to the benefit of potential investors. The International Capital Market Association’s (ICMA) guidelines^4^ standardize the specificities of green, sustainability and social bonds, e.g., in terms of eligible projects, reporting on the allocation of proceeds, and the use of external assurance such as certification, ultimately enhancing market transparency and disclosure. Over time, these guidelines have been updated according to the best practices, boosting market certainty and credibility against the risks of sustainability-washing. The success of sustainable debt securities has also been favored by policy initiatives and measures targeting sustainable finance, both directly and indirectly. Several countries have introduced mechanisms to facilitate and support green bond issuance, including guidelines, frameworks and regulations (Huang and Xu, 2024). In the context of its Sustainable Finance Strategy,^5^ in 2021 the European Commission presented a proposal for a regulation on a voluntary European green bond standard,^6^ which entered into force in December 2024 (see Fatica (2025) for a discussion of the European standard and its implications for the green bond market). More indirectly, other environmental and sustainability-oriented policies, such as environmental taxes, may motivate companies to prioritize environmentally friendly production processes and activities, and change their funding choices accordingly. At the same time, investor demand for non-conventional bonds has been on the rise, amid growing stakeholder awareness of sustainability issues (Rossi et al., 2019). At the global level, institutional investors have increased their engagement in responsible investment and explicit ESG strategies (Atta-Darkua et al., 2022) that involve offering a wider variety of sustainable products. A paradigmatic example is that of pension funds, whose long-term perspective is naturally aligned with the time horizon in the sustainability discourse (Hoepner and Schopohl, 2020). In July 2022, the European Central Bank announced the incorporation of climate change considerations into the purchases of corporate sector securities by the Eurosystem under the corporate sector purchase program (CSPP) and the pandemic emergency purchase program (PEPP).

In this paper, we aim to shed light on the determinants of raising finance through GSS bonds. We build a large international sample of conventional and non-conventional corporate bond issues between 2014 and 2022. First, we document a remarkable growth of the GSS market segment in the most recent years, spurred by the public commitment to financing a sustainable recovery after the COVID-19 pandemic. In the aftermath of the pandemic outbreak, volumes of social and sustainability bonds have overshadowed issuances of green bonds. After merging the bond data with issuers’ balance sheet information, we model corporate choices of bond instruments using a multinomial logit.

We focus on the countries’ stance towards sustainability, including the issue of sovereign GSS bonds, as well as on issuers’ relevant features, such as their broader sustainability commitment, the sector where they operate and previous access to the GSS market. We find evidence that corporations in countries with better environmental, social and governance (ESG) performance and with previous sovereign GSS issues are more likely to issue green and social bonds. As for the issuer features, we find that only the environmental and the governance components of the ESG score seem to have an effect on the probability of issuing green and social bonds, respectively. Sectoral considerations are important: companies engaging in green activities or in activities that are in transition towards sustainability are more likely to tap the GSS segment. This suggests that, consistent with their UoP, GSS bonds are more likely used by sectors that are already sustainable, or desire to become so, possibly also as a signaling device. Previous experience in the GSS segment, especially if accompanied by the use of costly external assurance, is also an important driver of corporate issues of non-conventional bonds.

By shedding light on the motivations for raising finance using new debt instruments with sustainable labels, our paper contributes to the literature on sustainable capital markets. While the sustainable bond literature has not established a comprehensive theory on issuer profiles and issuance determinants yet, different arguments have been suggested as rationale for corporate green issues. In Daubanes et al. (2021) green securities act as a commitment device towards climate-friendly investment. As such, they are used to signal to investors the profitability of green projects where managerial incentives play a crucial role. Barbalau and Zeni (2022) show that project-based securities, such as green bonds, provide the optimal design to correct for moral hazard issues in a principal-agent setup where reported green outcomes can be manipulated without costs. Empirical papers modeling issuance choices are equally scant and focused on green bonds only. Cicchiello et al. (2022) document that European firms with a higher portion of long-term debt and with higher female representation on boards have a higher probability of issuing a green bond. Lin and Su (2022), in a sample of Chinese issuers, examine the role of bonds’ specific characteristics, issuers’ financial features, and institutional conditions in influencing the choice of green issuance. More recently, Guesmi et al. (2025) find that firms exposed to climate change are more likely to issue green bonds. This relationship is primarily motivated by the firms’ desire to hedge against physical and regulatory risks rather than to capitalize on new climate-related opportunities. These findings are consistent with the survey results in Sangiorgi and Schopohl (2023), who report that the desire to curb climate change, together with reputational benefits and the market signaling power of green bonds, are the main motives for green bond issuance. Bedendo et al. (2023) find that previous commitment to environmental action is an important driver of green bond issues in the banking sector. Dutordoir et al. (2024) find an innovative path-dependence, with patent-strong companies rather pursuing R&D related to eco-innovations, and at the same time being more likely to offer green bonds. While these papers offer useful insights about issuance of green bonds, they are based on small sample sizes (i.e., one country, only issuers with at least a green bond). This translates into sometimes contrasting results and limits the possibility to generalize and extrapolate their findings and conclusions. In contrast, we use a comprehensive sample of corporate bond issuers, including state-owned firms, which allows us to take an international perspective on the rationale for accessing GSS issues. Moreover, we model the issuance of all non-conventional UoP bonds, i.e., green, sustainability and social, in a systematic way.

More broadly, our paper relates to the works that investigate access to the bond market. An extensive literature has investigated the use of fixed income securities in the context of corporate financing choices (Jung et al., 1996, Denis and Mihov, 2003, Erel et al., 2012), or as an alternative to bank credit (Morellec et al., 2015, Chen et al., 2020). Theoretical works have emphasized the role of agency costs and asymmetric information issues (Diamond, 1991, Bolton and Freixas, 2000, Boot and Thakor, 2000). Yet, the standard corporate finance literature treats debt securities as homogeneous instruments, or considers only differences stemming from their financial features. For instance, Gomes and Phillips (2012) analyze differences between public and private placements, and Badoer and James (2016) analyze the determinants of issuing debt securities with different maturities. We contribute to this strand of the literature by highlighting the role of sustainability factors and considerations in corporate finance choices.

The rest of the paper is organized as follows. Section 2 formulates the main hypotheses in the light of the relevant literature. Section 3 presents our data, the main trends for the non-conventional bonds in our sample, and our empirical framework. Section 4 illustrates the main results and Section 5 provides some additional evidence. Finally, Section 6 concludes.

## 2 Related literature and hypotheses development

In this section, we discuss different motives for corporate borrowers to raise funds in the sustainable bond segment as suggested by the related literature, and develop the hypotheses that will be tested in the empirical part of the paper.

The public stance towards sustainability plays an important role in shaping firms’ economic and financial choices, both directly and indirectly. More stringent legislation that favors corporate social responsibility and sustainability might indeed impose specific requirements on firms and create regulatory risks for non-compliance. The case of climate and environmental policy is emblematic. Putting a price on carbon decreases the value of carbon-intensive firms (Bolton and Kacperczyk, 2023), and could reduce their ability to access external finance (Nguyen and Phan, 2020), ultimately affecting their capital structure (Ginglinger and Moreau, 2023). Moreover, fragmented and uncoordinated policies favor regulatory arbitrage (Bartram et al., 2022).

Climate transition risk translates into higher costs of debt, both for bank credit (Fard et al., 2020, Delis et al., 2024, Ivanov et al., 2024) and in the conventional bond market (Seltzer et al., 2022). In a context where a favorable policy stance increases the relative returns of sustainable activities, resorting to the sustainable bond segment might be part of broader corporate strategies to avoid the financial penalties stemming from investors’ reactions to transition risks. Public policy may also incentivize the use of sustainable securities by directly subsidizing the costs for issuers and/or alleviating the tax burden on investors. The use of tax incentive schemes has been identified as an important determinant of the development of the US municipal bond market (Baker et al., 2022).^7^ In addition, public actors may support market certainty and integrity by establishing and implementing principles and standards for sustainable securities to help investors monitor and verify the actual sustainability effectiveness of their investments. This is the aim of the standard for a European green bond, introduced with a regulation and effective from December 2024.^8^

Another potential driver comes from governments, which, as borrowers in fixed income markets, can promote the development of the sustainable debt segment by directly issuing this type of security. Cheng et al. (2024) show that sovereign green bond issuances are followed by a more numerous and larger private green issuances, as they foster best practices in reporting and verification and improve market liquidity. More broadly, other papers have documented how sovereign bonds promote the development of the corporate bond market, for instance by reducing borrowing costs (Shiller, 1994, Flannery et al., 2023, Li et al., 2023). This evidence suggests that the public policy stance towards sustainability may affect the issuance of green, sustainability and social bonds. Therefore, we can formulate the following hypothesis:

*HP 1: Countries’ sustainability stance affects the propensity of issuing green, sustainability and social bonds.*

If the use of GSS securities is instrumental in pursuing broader corporate sustainability engagement, we expect it to be associated with a better overall sustainability profile, or efforts to achieve one. While measurement divergence increases the noise of their information signal (Berg et al., 2022), ESG metrics provide a useful tool to identify better performing companies on the environmental, social and governance dimensions (Ferrell et al., 2016, Liang and Renneboog, 2017). In the context of the bond market, Apergis et al. (2022) find that issuance costs are lower for firms with better ESG ratings, while the benefits of better ESG performance on secondary markets seem to materialize only in crisis times (Amiraslani et al., 2023).

The feature of GSS bonds as use-of-proceeds securities implies that the funds raised are used to finance new or existing eligible projects that provide sustainability benefits. Empirical evidence shows that green bond financing has a real effect, in that green issuers appear to reduce their carbon footprint (Flammer, 2021, Fatica and Panzica, 2021, ElBannan and Löffler, 2024), possibly by engaging more in green innovation after raising non-conventional bond finance (Dong et al., 2024). As described in Section 1, categories of expenditure associated with the use of green securities, and partly of sustainability bonds, are predominantly related to climate change mitigation purposes, including notably renewable energy and energy efficiency. Hence, arguably, non-conventional bond financing would be the preferred choice not only by firms that are already engaged in green activities, but also by companies that need to fund climate-related investment to decarbonize their operations (Guesmi et al., 2025). Given the current limitations in corporate sustainability reporting and disclosure, the identification of sustainable vs harmful activities still largely relies on the standard sectoral classifications. On the investor side, sustainability-conscious investment strategies have long been based on company industries before ESG-based allocation became popular. For example, the strategy of excluding stocks of companies involved in harmful or controversial activities, the so-called sin stocks (Fabozzi et al., 2008, Hong and Kacperczyk, 2009), is widely followed by socially responsible investors.^9^ Similarly, policy initiatives aimed at facilitating sustainable investments and channeling financial resources towards sustainable activities are generally based on sectoral classifications. In a context where firm-level and project-level information asymmetries are still very high, sustainable securities may serve as a signaling device of companies’ sustainability commitments (Daubanes et al., 2021, Barbalau and Zeni, 2022), thus boosting their credentials and attracting investor attention. The recent empirical literature on green bonds is consistent with a signaling mechanism being at play in driving the greenium, i.e., a lower yield on green compared to conventional bonds (Flammer, 2021, Fatica et al., 2021, Zerbib, 2019),^10^ and behind the improved corporate carbon performance after green bond issues (Fatica and Panzica, 2021, ElBannan and Löffler, 2024), and the positive stock market reaction to green bond issuance announcements (Tang and Zhang, 2020).

The link that GSS securities have with corporate investment decisions, along with the young age of the sustainable segment, makes GSS bonds likely to suffer from higher information asymmetries than conventional fixed income instruments. Theoretically, asymmetric information is a pervasive issue between companies and suppliers of funds (Akerlof et al., 1970), but reputable information, certification and even voluntary disclosure, when costly, may become valuable signals to market participants (Riley, 1979). On the issuer side, the additional requirements – such as those related to the preparation of the bond framework, reporting, and use of external assurance – may act as a barrier to entry into the market, not least by lengthening the issue process compared to conventional bonds (Sangiorgi and Schopohl, 2023). In this context, repeated GSS issuance allows issuers to learn and align with best market practices, while enabling investor scrutiny of the borrower’s sustainability credentials. Likewise, the use of costly assurance mechanisms, such as certification and external verification, strengthens the signal from issuing in the sustainable bond segment, thus, in turn, bolstering the credibility of the issuers’ commitment towards sustainability. Experience in the green bond market and external assurance has been found to translate into an additional price premium for green bonds (Fatica et al., 2021, Flammer, 2021).

The discussion above leads us to formulate the following hypotheses:

*HP 2A: Issuers with higher levels of sustainability engagement, as reflected in ESG ratings, have a higher propensity to issue GSS bonds.*
*HP 2B: Issuers engaging in sustainable activities or that need to transition towards sustainability have a higher propensity to issue GSS bonds.*
*HP 2C: Issuers that have already committed to green, sustainability and social signals have a higher propensity to issue GSS bonds.*

## 3 Data and empirical model

### 3.1 Data

We draw bond data from Dealogic DCM, a leading provider of information on global fixed income issuances. DCM reports detailed bond characteristics at the tranche level. We initially select all bond tranches issued in the period 2014–2022, corresponding to approximately 250,000 bonds. Information about the bond type allows us to distinguish between conventional bonds and non-conventional securities, notably green, sustainability and social bonds.

DCM does not provide a separate category for sustainability-linked bonds, a type of debt security introduced in 2017 embedding contingencies ensuring commitments to specific outcomes (e.g., the amount of GHG absolute emissions or the number of jobs created in a host community). We retrieve sustainability-linked bonds (SLBs) from Bloomberg and after merging the data with our DCM sample, in which SLBs are mainly considered as conventional bonds, we classify these bonds as green, sustainability or social based on their main objective by looking at the key performance indicators (KPIs).^11^

We match bond-level information with company-level financial data. We perform the matching by retrieving the company’s and bond’ identifiers. We use a manual matching procedure based on firms’ names when company identifiers are not available. Balance sheet data are from the Orbis database provided by Moody’s Bureau Van Dijk. We only retain bond issuers with valid financial statements. Given the specificities, in both frequency and amounts raised, in their access to the bond market, including on the GSS segments, we exclude issuers that are classified as banks, financial institutions, or report financial activities as their main sector.^12^ We also exclude governmental borrowers. Hence, we retain corporate issuers, including state-owned companies. The final sample includes 7220 unique corporate issuers located in 102 countries worldwide for a total of 60,967 issuer-year observations.^13^ Finally, we complement our dataset with country-level data from the World Bank’s World Development Indicators database.

### 3.2 Empirical approach

To shed light on the choice of debt issuance and the different bond types, we estimate a multinomial logit model that accommodates for the presence of multiple alternatives. The general formulation of the econometric model is as follows:

(1) $$Prob(B=0,1,2,3,4)=F(\beta_{B}X)$$

where B is a discrete dependent variable which can take five values: zero if the issuer issues no bonds in the year, one if it issues conventional bonds only, two if it issues green bonds, three if it issues sustainability bonds, four if it issues social bonds.^14^ In case a borrower in the same year issues different types of bonds, we attribute the choice to the issue category corresponding to the larger amount.^15^F(⋅) is the logistic distribution; $\beta_{B}$ is a vector of coefficients for outcome B while X is a vector of explanatory variables. In all our specifications, following previous literature on corporate access to bond markets, we include macroeconomic controls and firm-level variables. To capture global market conditions, we include the variable VIX, the implied volatility on options on the S&P500 Index. As domestic explanatory variables, we consider Inflation and GDPgrowth in the country of incorporation of the borrower. These variables capture the business cycle fluctuations and idiosyncratic shocks, which may affect borrowers’ propensity to raise debt finance. We account for the presence of state-owned companies in our sample by defining the dummy variable Publicissuer that takes unit value if the issuer is a public interest company, and zero otherwise. To control for the borrower’s financial conditions we include the following variables: Size, as the natural logarithm of the book value of total assets (in millions of Euro); Leverage, defined as the ratio of debt to the book value of total assets; ROA, as the ratio between operating income before depreciation and the book value of total assets. Furthermore, we include Age, defined as the difference between the current year and the year when the company entered the market, and Listed, a dummy variable that equals one if the issuer is listed in a public market, and zero otherwise. These can be important predictors of companies’ capacity to access the debt market. To avoid simultaneity issues, all explanatory variables are lagged by one year. Finally, we add year × geographic area fixed effects to capture differences in the propensity to issue bonds due to time-varying factors at the regional level. We calculate the standard errors allowing for clustering of observations at the issuer level. The equation is estimated using a panel of yearly observations for the sample of issuers that have issued at least one bond in the period 2014–22. This baseline model is then augmented with additional country- and issuer-level variables to test our main hypotheses. Table A.2 in the Appendix tabulates the definitions of all variables used in the empirical model.

### 3.3 Sample overview

**Fig. 1:**
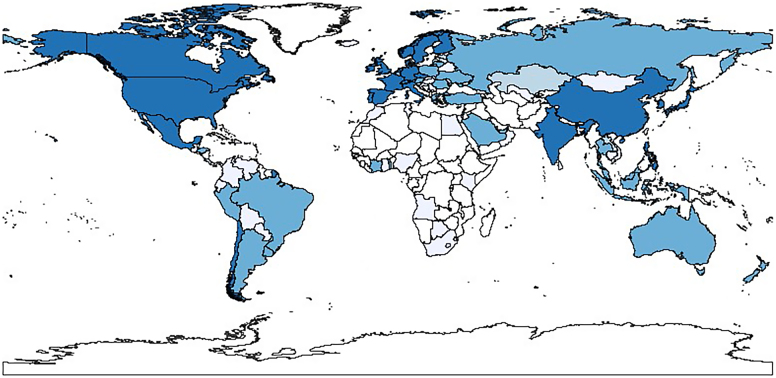
Geographic distribution of green, sustainability and social bond issuers. The map visualizes the cumulated worldwide corporate GSS bond issuance over the period 2014–22. The color coding indicates relative intensity, i.e., the darker the blue, the higher the volume of GSS bonds that have been issued. White signifies that no GSS bonds have been issued.

**Fig. 2:**
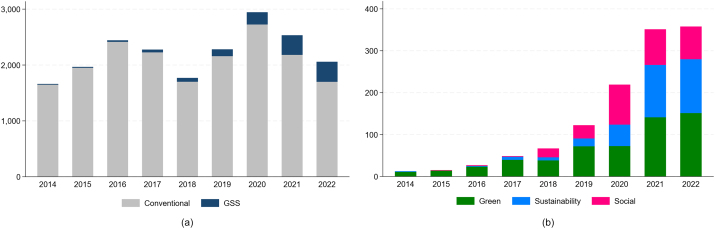
Total Amount of conventional and GSS bond issues over time. The left chart in panel (a) displays the total amount (in billions of euros) of conventional and GSS bond issues by year. The chart in panel (b) displays the total amount (in billions of euros) of green, sustainability and social bond issues by year. (For interpretation of the references to colour in this figure legend, the reader is referred to the web version of this article.)

**Fig. 3:**
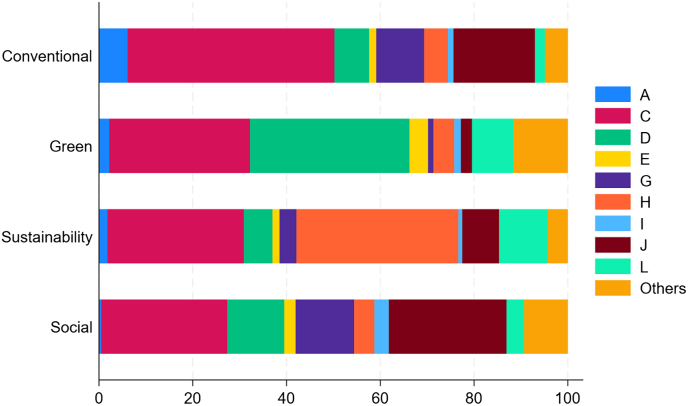
Breakdown of conventional, green, sustainability and social bond volumes by sector of the borrower. NACE sections are A (Agriculture, forestry, and fishing), C (Manufacturing), D (Electricity, gas, steam, and air conditioning supply), E (Water supply, sewerage, waste management), G (Wholesale and retail trade, repair of motor vehicles and motorcycles), H (Transportation and storage), I (Accommodation and food service activities), J (Information and communication), L (Real estate activities), and Others including all other sectors. (For interpretation of the references to colour in this figure legend, the reader is referred to the web version of this article.)

Although the GSS segment is still a niche of the bond market, issues of non-conventional bonds have increased at a remarkable pace. Fig. 2 Panel A shows the annual issuance of conventional and GSS bonds in the period 2014–22. Growth is particularly pronounced in 2020–22, the more recent sample years. In Panel B we break down the aggregate GSS issue into its three components: green, sustainability and social bonds. While green bonds were the predominant instrument in the early years, sustainability and social bonds have gained significant ground in the non-conventional debt market since the Covid-19 crisis. As is apparent, unlike conventional bond volumes, which have contracted, GSS issues have not been affected by the recent geo-political and economic tensions. Fig. 3 reports the breakdown of issues by the main NACE sector of the borrower. Manufacturing firms account for the largest share of issued volumes. Borrowers operating in electricity, gas, steam, and air conditioning supply and real estate activities are large green bond issuers, whereas the transportation and storage sectors have the lion’s share of sustainability bond issues. Social bonds are predominantly issued by companies in the information and communication sectors. Fig. 1 maps the countries with GSS borrowers in our sample, with darker shades corresponding to larger issued amounts. The map indicates a widespread coverage, with non-conventional debt being raised particularly in Europe, North America and South Asia. Table 1 details the 15 countries with the largest issues, in terms of number of contracts (Panel A) and amount issued (Panel B). Significant cross-section heterogeneity exists at the extensive vs. the intensive margin. In terms of number of issues, the highest frequency of GSS contracts is recorded in Sweden, the Netherlands, Japan, Finland, and Italy. Further breakdown by specific sustainable bond type shows that the number of sustainability and social securities is still limited. The amounts issued offer a complementary picture of the GSS market. There are striking differences between EU and non-EU countries, with a larger amount, on average, of GSS bonds (in percentage of the total amount issued in each jurisdiction) issued by companies located in EU countries. The largest non-conventional issuers are located in France, Italy (around 16 %), the Netherlands, and Sweden (around 14 %). By contrast, large bond borrowers in the United States and China rely much less on GSS securities than they do on conventional debt instruments.

**Table 1:** Bond issuance for the top 15 issuing countries. Column 2 reports a dummy variable EU, which is equal one if a country is part of the European Union. Column 3 refers to conventional bonds, column 4 to GSS bonds, column 5 to green bonds, column 6 to sustainability bonds, and column 7 to social bonds. Panel A shows the frequency (in %) of the dependent variable Issuetype taking a given value in the issuer-year sample (i.e., the column *Conv* shows the frequency of conventional bond issues in the issuer-year panel). Panel B shows the volumes issued by bond type as a % of total bond issues in each jurisdiction.

Country	EU	Conv	GSS	Green	Sust	Soc
Panel A: Issuetype Number (%)						
USA	0	26.01	1.36	0.86	0.22	0.28
CHN	0	35.70	3.05	2.71	0.06	0.29
JPN	0	35.95	5.74	2.85	1.45	1.43
SWE	1	32.39	11.44	10.26	0.76	0.42
KOR	0	28.40	3.29	1.61	0.84	0.84
FRA	1	28.22	3.32	1.71	1.31	0.31
NLD	1	28.77	11.40	7.54	1.75	2.11
NOR	0	24.18	4.09	3.65	0.45	0.00
DEU	1	27.40	3.59	2.67	0.76	0.15
ITA	1	23.31	5.14	2.97	1.71	0.46
GBR	0	21.44	2.18	1.17	0.35	0.66
ESP	1	22.05	5.06	3.76	1.04	0.26
IND	0	22.08	1.74	1.69	0.05	0.00
FIN	1	24.62	5.68	4.92	0.57	0.19
CHE	0	30.60	1.94	1.94	0.00	0.00
Panel B: Total amount (%)						
USA	0	96.72	3.28	0.97	0.16	2.15
CHN	0	95.38	4.62	3.51	0.07	1.04
JPN	0	92.70	7.30	2.83	2.80	1.67
SWE	1	86.04	13.96	12.08	1.52	0.35
KOR	0	88.85	11.15	4.28	4.74	2.13
FRA	1	83.40	16.60	4.13	12.10	0.37
NLD	1	85.32	14.68	8.33	3.19	3.17
NOR	0	89.61	10.39	9.08	1.30	0.00
DEU	1	94.50	5.50	4.30	1.07	0.13
ITA	1	83.46	16.54	5.16	10.50	0.88
GBR	0	96.06	3.94	2.24	0.69	1.00
ESP	1	87.30	12.70	8.17	2.87	1.66
IND	0	91.48	8.52	8.38	0.13	0.00
FIN	1	89.20	10.80	9.48	0.86	0.46
CHE	0	95.00	5.00	5.00	0.00	0.00

Table 2 shows the summary statistics of the variables used in our empirical analysis. All variables are winsorized at the 1 % and 99 % percentiles of their empirical distributions. On average, the issuers in our sample are very large, established companies with an average age of 36 years. More than half of the issuers in our sample are listed, while around 18 % are state-owned.

**Table 2:** Summary statistics. This table provides summary statistics for the main sample variables. All control variables are lagged by one period and are winsorized at the 1 % and 99 % percentiles of their empirical distributions. Variables are defined in Table A.2. The sample period is from 2014 to 2022.

Variable	N	Mean	St. dev.	P.le 50	P.le 5	P.le 95
VIX	60967	18.11	5.80	16.60	10.15	29.46
Inflation	60967	2.42	3.56	1.79	−0.35	7.50
GDP growth	60967	2.46	3.28	2.29	−3.28	7.43
Public issuer	60967	0.18	0.38	0.00	0.00	1.00
Size	60967	15.69	2.29	15.57	12.08	19.29
Leverage	60967	0.69	0.21	0.68	0.35	1.00
ROA	60967	0.02	0.05	0.02	−0.06	0.11
Age	60967	36.84	35.79	24.00	4.00	111.00
Listed	60967	0.56	0.50	1.00	0.00	1.00

## 4 Results

### 4.1 Baseline results

Before testing our hypotheses, in Table 3 we present the results from estimating the baseline model for bond type choice in Eq. (1). Model (1) shows the estimates from a multinomial logit regression in which the outcome is 0 if no bond has been issued in that year, 1 if only a conventional bond has been issued, and 2 if any of the GSS bond types have been issued. The base outcome is not issuing any bonds in the given year. Hence, the estimates in column II can be interpreted as the impact of the different variables on the probability of issuing a GSS bond vis-à-vis not issuing any bonds at all. Looking at column I, we notice that higher stock market volatility, inflation, and GDP growth reduce the probability of issuing conventional bonds. Domestic economic conditions, as captured by inflation and GDP growth, have a similar impact on the issue of GSS bonds, while stock market uncertainty does not seem to discourage access to the sustainable bond segment (column II).

**Table 3:** Propensity to issue non-conventional bonds: this table reports the results of a multinominal logit model for the choice of bond type. In model (1) the dependent variable equals zero (one) [two] in years in which a firm issues no bonds (conventional bonds only) [non-conventional bonds]. In model (2) the dependent variable equals zero (one) in years in which a firm issues no bonds (conventional bonds only); it equals two (three) [four] in years in which a firm issues green bonds (sustainability bonds) [social bonds]. Control variables are defined in Table A.2. All models include year × geographic area fixed effects. Standard errors, robust to heteroscedasticity, are clustered at the issuer level; t-stats are reported in parentheses and significance levels are reported as follows: ***p<0.01, **p<0.05, *p<0.1.

Outcome	(1)		(2)			
	Conv	Non-conv	Conv	Green	Sust	Soc
	I	II	III	IV	V	VI
VIX	−0.0285***	0.0108	−0.0285***	−0.0073	−0.0111	0.3072
	(0.0043)	(0.0196)	(0.0043)	(0.0217)	(0.0456)	(0.2176)
Inflation	−0.0125***	−0.0637***	−0.0125***	−0.0594***	−0.0837***	−0.0546*
	(0.0033)	(0.0117)	(0.0033)	(0.0131)	(0.0302)	(0.0283)
GDP growth	−0.0146**	−0.0262*	−0.0146**	0.0358**	−0.1690***	−0.1270***
	(0.0064)	(0.0149)	(0.0064)	(0.0165)	(0.0337)	(0.0309)
Public issuer	0.1876***	0.9377***	0.1875***	0.7218***	1.3772***	1.3959***
	(0.0449)	(0.1083)	(0.0449)	(0.1279)	(0.2012)	(0.2457)
Size	0.0109	0.1732***	0.0110	0.1712***	0.1539***	0.2100***
	(0.0069)	(0.0193)	(0.0069)	(0.0206)	(0.0417)	(0.0603)
Leverage	0.0982	−0.5641***	0.0981	−0.5670***	−0.5183	−0.7459
	(0.0679)	(0.1992)	(0.0679)	(0.2137)	(0.5344)	(0.5381)
ROA	2.2305***	3.0238***	2.2313***	3.0649***	1.8274	4.2237*
	(0.2270)	(0.6760)	(0.2270)	(0.7206)	(2.0152)	(2.2084)
Age	0.0012***	0.0015	0.0012***	−0.0007	0.0059***	0.0044**
	(0.0004)	(0.0010)	(0.0004)	(0.0013)	(0.0016)	(0.0019)
Listed	0.1007***	−0.1441*	0.1008***	−0.0897	−0.5698***	0.1019
	(0.0311)	(0.0869)	(0.0311)	(0.1051)	(0.1656)	(0.1781)
Observations	60,967	60,967	60,967	60,967	60,967	60,967
of which Y=1	16,706	16,706				
of which Y=2	1,601	1,601				
of which Y=1			16,706	16,706	16,706	16,706
of which Y=2			1,080	1,080	1,080	1,080
of which Y=3			277	277	277	277
of which Y=4			244	244	244	244
Pseudo R-squared	0.0340	0.0340	0.0359	0.0359	0.0359	0.0359
Year × Geo FE	Yes	Yes	Yes	Yes	Yes	Yes

Some important differences in the propensity to issue GSS and conventional securities emerge when we consider the issuer’s characteristics. The coefficient of Size in column II is positive and significant at the 1 % level, suggesting that larger issuers are more likely to raise GSS debt than not issuing bonds at all. This is consistent with the fact that larger borrowers are less subject to information asymmetries and have easier access to alternative funding sources (Frank and Goyal, 2009). By contrast, firm size does not affect the issue of conventional bonds in a statistically significant way. The coefficients of Leverage suggest that lower indebtedness favors the issuance of GSS bonds but not conventional bonds. We also find that, holding other variables constant, being listed decreases the probability of issuing a GSS bond by 4 percentage points. This implies that raising public equity is not per se an enabler for accessing the non-conventional debt segment. In contrast, listed and older issuers are more likely to issue conventional bonds. Public and more profitable issuers do not display a clear preferential pattern for different types of bonds, compared to not issuing fixed income at all.

Next, we split the GSS category into its components, and take to the data the full baseline model in Eq. (1) with four bond types as described in Section 3.2. Table 3 reports the coefficient estimates for the choice of conventional bonds (column III), green (column IV), sustainability (column V) and social bonds (column VI).^16^ Focusing on the main differences, we notice that, in contrast to the other GSS securities, the issuance of green bonds seems to be pro-cyclical. This result might reflect the surge in the use of sustainability and social bonds in the aftermath of the COVID-19 pandemic. The profiles of issuers of GSS bonds show some differences as well. All three types of non-conventional bonds are more likely to be used by public borrowers. Based on the marginal effects, everything else being equal, state-owned companies are twice as likely as non public issuers to issue a green bond (2.7 % as opposed to 1.5 %), and, about half as likely to issue a sustainability bond or a social bond (1.0 % as opposed to 0.3 %). The probability of issuing green bonds is higher for less leveraged and more profitable firms. This evidence is in line with previous studies exploring the relationships between the use of corporate bond financing and firms’ characteristics (Denis and Mihov, 2003). We also observe that leverage and profitability do not seem relevant for the choice of sustainability and social bonds. Overall, these results point to some differences in how macroeconomic and firm-specific factors influence the choice of debt instruments, even within the GSS category. However, with the sustainable debt segment still far from reaching maturity, it is arguably too early to identify structural patterns and to define a specific profile of sustainable borrowers. Further market development, particularly for the sustainability and social segments, will possibly enable a better characterization of the drivers of GSS market access and of the possible differences with respect to the conventional segment.

### 4.2 The role of country’s sustainability characteristics and policies

In this section, we test whether the likelihood of issuing corporate GSS bonds is affected by the domestic policy stance and general progress towards sustainability at the country level (Hypothesis 1). Following a global call for tackling pressing development and climate issues, many countries in the last decade have promoted sustainable policies and strengthened their commitment to stricter environmental and sustainability targets. Still substantial differences persist across countries. Our international sample allows us precisely to leverage this heterogeneity in countries’ policies and preparedness. First, to measure countries’ climate policies we include in our baseline model the Notre-Dame Global Adaptation Index, ND-GAIN, a measure of a country’s readiness to face climate change.^17^ Second, in line with previous works (Hoepner et al., 2016, Capelle-Blancard et al., 2019), we consider the environmental, social and governance indicators provided by the World Bank (WB). For each category of the WB indicators, we use principal component analysis to pin down the variables that account for most of the variance in the corresponding sustainability areas. We include the first component of each group of indicators, denoted as CountryE−pillar, CountryS−pillar, and CountryG−pillar, respectively, in the multinomial logit model as more comprehensive proxies for countries’ sustainability profile.

Results are reported in Table 4. All model specifications, in addition to issuer characteristics, include both macro-financial covariates and year × geographical area fixed effects. This allows us to identify the effect of the specific sustainability characteristics, while controlling for domestic macro-financial conditions and time-varying factors common to geographical areas that affect corporate bond issuance choices. The estimates in Panel A show that corporate borrowers in countries with better climate preparedness have a higher likelihood to issue green and social bonds. Panel B shows that the domestic environmental and governance pillars are negatively associated to the likelihood of issuing a conventional bond. In contrast, green bonds are more likely issued by borrowers located in countries with higher environmental and governance pillars (column II). In a similar way, column IV shows that the country’s social pillar affects positively the issue of social bonds. The effect is only marginally statistically significant though.

**Table 4:** Propensity to issue non-conventional bonds and countries’ stance towards sustainability. In all panels the dependent variable equals zero (one) in years in which a firm issues no bonds (conventional bonds only); it equals two (three) [four] in years in which a firm issues green bonds (sustainability bonds) [social bonds]. In panel A ND−GAIN is a measure of a country’s preparedness for climate change. In panel B CountryE−pillar 1st component of the PCA on the country environmental indicators; CountryS−pillar 1st component of the PCA on the country social indicators; CountryG−pillar 1st component of the PCA on the country governance indicators. In panel C SovereignGSS is a dummy variable equal to one if sovereign GSS bonds have been issued in the jurisdiction where the borrower is incorporated, and zero otherwise. All models include the set of control variables reported in Table 2 and defined in Table A.2, and year × geographic area fixed effects. Standard errors are robust to heteroscedasticity and clustered at the issuer level; t-stats are reported in parentheses and significance levels are reported as follows: ***p<0.01, **p<0.05, *p<0.1.

Outcome	Conv	Green	Sust	Soc
	I	II	III	IV
Panel A				
ND-GAIN	−0.0002	0.0384***	0.0127	0.0390***
	(0.0020)	(0.0068)	(0.0193)	(0.0125)
Observations	60,911	60,911	60,911	60,911
Pseudo $R^{2}$	0.0368	0.0368	0.0368	0.0368
Macro & firm controls	Yes	Yes	Yes	Yes
Year FE × Geo FE	Yes	Yes	Yes	Yes
Panel B				
Country E-pillar	−0.2455***	0.6151**	−0.3086	−1.2282
	(0.0515)	(0.2391)	(0.3978)	(0.8528)
Country S-pillar	−0.0798***	0.2351	0.1136	0.5720*
	(0.0309)	(0.1439)	(0.3320)	(0.3448)
Country G-pillar	−0.0867*	0.8483***	−0.2810	−0.7982
	(0.0475)	(0.1592)	(0.3425)	(0.5469)
Observations	31,209	31,209	31,209	31,209
Pseudo $R^{2}$	0.0356	0.0356	0.0356	0.0356
Macro & firm controls	Yes	Yes	Yes	Yes
Year FE × Geo FE	Yes	Yes	Yes	Yes
Panel C				
Sovereign GSS	−0.3772***	0.1942*	0.0827	0.3440*
	(0.0546)	(0.1158)	(0.1966)	(0.1482)
Observations	60,967	60,967	60,967	60,967
Pseudo $R^{2}$	0.0370	0.0370	0.0370	0.0370
Macro & firm controls	Yes	Yes	Yes	Yes
Year FE × Geo FE	Yes	Yes	Yes	Yes

In addition to specific sustainable policies and regulations, public authorities may also directly raise finance on the sustainable bond market for green, sustainability or social projects. This might favor corporate issues thanks to better liquidity conditions and the creation of more sustainable investment opportunities. We introduce the variable SovereignGSS that is equal to one if an issuer is located in a country in which the sovereign has previously issued any of the GSS bonds, and zero otherwise. Panel C shows that the probability of issuing a green bond and a social bond is higher in countries with previous sovereign GSS bond issues. This corroborates the results in Cheng et al. (2024) who find that sovereign green bond issues act as a catalyst for the domestic market development. Overall, these findings support our first hypothesis and corroborate prior results on the key role of public policy initiatives in influencing firms decisions to factor in sustainability in their operations (Nguyen and Phan, 2020, Bolton and Kacperczyk, 2023).

### 4.3 The role of issuers’ sustainability profile

In this section, we explore whether borrowers’ sustainability performance and commitment are relevant for the decision to issue a specific type of bond (Hypotheses 2A–2C).

In the last decade, financial market participants have devoted increasing attention to ESG considerations. ESG ratings have been identified as important factors influencing financial performance (Lins et al., 2017), corporate leverage (Asimakopoulos et al., 2023), as well as the cost of funding (Liu et al., 2024), even in the context of established relationship lending (Houston and Shan, 2022). Hence, we first consider ESG scores as a measure of borrowers’ sustainability performance. We retrieve the scores from Refinitiv Eikon.^18^ We alternatively include the aggregate ESG score and its individual components as additional covariates into Eq. (1). The results are reported in Table 5.

**Table 5:** Propensity to issue non-conventional bonds and ESG scores. In all panels the dependent variable equals zero (one) in years in which a firm issues no bonds (conventional bonds only); it equals two (three) [four] in years in which a firm issues green bonds (sustainability bonds) [social bonds]. ESG is the issuer ESG score (panel A). Escore is the issuer environmental score (panel B), Sscore is the issuer social score (panel C), and Gscore is the issuer governance score (panel D). All models include the set of controls reported in Table 2 and defined in Table A.2, and year × geographic area fixed effects. Standard errors are robust to heteroscedasticity and clustered at the issuer level; t-stats are reported in parentheses and significance levels are reported as follows: ***p<0.01, **p<0.05, *p<0.1.

Outcome	Conv	Green	Sust	Soc
	I	II	III	IV
Panel A				
ESG	0.0002	0.0044	−0.0031	−0.0070
	(0.0011)	(0.0047)	(0.0071)	(0.0067)
Observations	16,302	16,302	16,302	16,302
Pseudo R-squared	0.0419	0.0419	0.0419	0.0419
Macro & firm controls	Yes	Yes	Yes	Yes
Year FE × Geo FE	Yes	Yes	Yes	Yes
Panel B				
E score	0.0004	0.0093**	−0.0013	0.0004
	(0.0010)	(0.0041)	(0.0064)	(0.0071)
Observations	16,302	16,302	16,302	16,302
Pseudo R-squared	0.0422	0.0422	0.0422	0.0422
Macro & firm controls	Yes	Yes	Yes	Yes
Year FE × Geo FE	Yes	Yes	Yes	Yes
Panel C				
S score	0.0002	0.0001	0.0022	−0.0033
	(0.0010)	(0.0038)	(0.0068)	(0.0062)
Observations	16,302	16,302	16,302	16,302
Pseudo R-squared	0.0417	0.0417	0.0417	0.0417
Macro & firm controls	Yes	Yes	Yes	Yes
Year FE × Geo FE	Yes	Yes	Yes	Yes
Panel D				
G score	−0.0004	0.0006	−0.0097	0.0148***
	(0.0010)	(0.0038)	(0.0066)	(0.0053)
Observations	16,302	16,302	16,302	16,302
Pseudo R-squared	0.0421	0.0421	0.0421	0.0421
Macro & firm controls	Yes	Yes	Yes	Yes
Year FE × Geo FE	Yes	Yes	Yes	Yes

The coefficient of the ESG score is not statistically different from zero for all types of bonds (Panel A). While better ESG performance seems associated with lower yields in the primary bond market for large listed US companies (Apergis et al., 2022), it is not directly linked to a higher propensity to issue in our sample of global issuers. A different picture emerges when we include the individual components of the ESG separately. A higher environmental score increases the probability of issuing green bonds (Panel B), whereas a higher governance score is associated with a higher likelihood of issuing social bonds (Panel D). These potentially contradicting results support the notion that the aggregation process of environmental, social and governance data affects the information content of the individual components (Berg et al., 2022). Also empirical studies examining the relationship between ESG scores and corporate debt structure often yield mixed findings (Sharfman and Fernando, 2008, Asimakopoulos et al., 2023).

Next, we explore the link between companies’ operations and their financing choices (Hypothesis 2B). To gauge firms’ current and potential degree of alignment with environmental sustainability, we rely on their sector of activity.^19^ We borrow the classification under the so-called ‘EU Taxonomy for Sustainable Activities’, a comprehensive and science-based categorization that identifies the extent to which an economic activity is environmentally sustainable, and under which conditions.^20^ The classification relies on the NACE (Nomenclature statistique des Activite economiques dans la Communaute Europeenne) version 2, the official industrial classification system used in Europe. Using the main sector of the issuers as reported in Orbis, we create the dummy variable Taxonomy, which takes value 1 if the issuer has a NACE included in the list of taxonomy-eligible sectors (the full list of sectors is provided in Table A.4 in Appendix), and zero otherwise. The results are reported in Table 6, Panel A. The estimates show that engaging in environment-friendly activities increases the likelihood to issue green bonds and also social bonds, while it has a statistically insignificant impact on the likelihood of using conventional and sustainability bonds.

**Table 6:** Propensity to issue non-conventional bonds and the issuer’s sector of activity. In all panels the dependent variable equals zero (one) in years in which a firm issues no bonds (conventional bonds only); it equals two (three) [four] in years in which a firm issues green bonds (sustainability bonds) [social bonds]. In panel A, Taxonomy is a dummy variable equal to one if the issuer’s sector is among the eligible sectors according to the EU taxonomy of sustainable activities. In panel B, Hard-to-abate is a dummy variable equal to one if the issuer’s sector is classified as hard-to-abate. In panel C, Transition is a dummy variable equal to one if the issuer’s sector is included in the list of transition sectors. All models include the set of control variables reported in Table 2 and defined in Table A.2, year × geographic area fixed effects and sector fixed effects. Standard errors are robust to heteroscedasticity and clustered at the issuer level; t-stats are reported in parentheses and significance levels are reported as follows: ***p<0.01, **p<0.05, *p<0.1.

Outcome	Conv	Green	Sust	Soc
	I	II	III	IV
Panel A				
Taxonomy	0.1038	0.3724**	−0.1508	0.6254**
	(0.0775)	(0.1743)	(0.3379)	(0.2843)
Observations	60,499	60,499	60,499	60,499
Pseudo R-squared	0.0472	0.0472	0.0472	0.0472
Macro & firm controls	Yes	Yes	Yes	Yes
Year FE × Geo FE	Yes	Yes	Yes	Yes
Sector FE	Yes	Yes	Yes	Yes
Panel B				
Hard-to-abate	0.1484**	0.4048	−0.8280	0.7699**
	(0.0736)	(0.2562)	(0.5831)	(0.3313)
Observations	60,499	60,499	60,499	60,499
Pseudo R-squared	0.0472	0.0472	0.0472	0.0472
Macro & firm controls	Yes	Yes	Yes	Yes
Year FE × Geo FE	Yes	Yes	Yes	Yes
Sector FE	Yes	Yes	Yes	Yes
Panel C				
Transition	0.1832***	0.5467***	−0.7008	0.5829*
	(0.0677)	(0.2074)	(0.5123)	(0.3050)
Observations	60,499	60,499	60,499	60,499
Pseudo R-squared	0.0473	0.0473	0.0473	0.0473
Macro & firm controls	Yes	Yes	Yes	Yes
Year FE × Geo FE	Yes	Yes	Yes	Yes
Sector FE	Yes	Yes	Yes	Yes

A set of industries that deserve special attention from the climate perspective are the energy-intensive industries, and among these the ones considered hard-to-abate (HtA). HtA activities, which include heavy-duty trucking, shipping, aviation, iron and steel, and chemicals and petrochemicals, use fossil resources as fuel or feedstock.^21^ In these activities, greenhouse gas (GHG) emissions are comparatively difficult to reduce using current technologies. Hence, HtA sectors face important transition risks given the strong decarbonization efforts required to meet the global climate goals, while their competitiveness and profit margins are eroded as energy costs rise. We define a Hard-to-abate dummy equal to one for HtA borrowers, and zero otherwise. Panel B in Table 6 reveals that, compared to borrowers operating in other sectors, hard-to-abate borrowers are more likely to issue ordinary bonds, by 2 percentage points. Within the GSS pool, we find a positive and statistically significant effect on the probability of issuing social bonds, but no impact for the choice of green or sustainability bonds, which are directly linked to environmental and climate-related use of proceeds, as documented in Section 1. These findings suggest limited capacity to tap the unconventional debt segment by these industries, but also potentially the possibility to leverage sustainability preferences by emphasizing social rather environmental and climate considerations, also from a signaling perspective (Daubanes et al., 2021).

In the context of the net-zero transition, an important contribution is made by activities that are currently characterized by high greenhouse gas emissions or other environmental impacts, but are moving towards environment-friendly performance levels over time. Transition finance refers precisely to funding transition-enabling private investments, such as those that provide green production methods or reduce the environmental footprint as much as possible, where green technologies are not available yet. As before, we use a NACE-based sectoral classification to pin down activities in transition. Then, we augment the baseline multinomial logit model with a Transition dummy, which takes unit value for transition borrowers, and zero otherwise. Panel C in Table 6 reports the estimates of the corresponding coefficient. We observe that operating in a transition sector is associated to a higher likelihood of issuing green and social bonds, versus issuing no bonds, but has no impact on the probability of using conventional and sustainability bonds. The small size of the impact presumably reflects the fact that all specifications, in addition to year × geographical area fixed effects, are saturated with sector fixed effects (at section level). Overall, in spite of the challenges for the econometric identification, this evidence suggests that real economy considerations are important motives to borrow on the GSS segment.

In a context where the high information asymmetries strengthen the effect of credible signals, sustainability commitment through the use of GSS securities might provide significant incentives to return to the same segment to reap the benefits of learning and reputation building, including because of external assurance (Hypotheses 2C). In Table 7 we explore the role of these market mechanisms in different ways. In Panel A we include Greenissuer, a dummy variable equal to one if the issuer in the previous years has issued a green bond, and zero otherwise. In a similar fashion, we define the dummy variables Sustainabilityissuer and Socialissuer for the other corresponding categories of GSS bonds, with which we augment the baseline multinomial logit. The corresponding coefficients in Columns II–IV are positive and highly statistically significant throughout. The relative size of the estimates indicates that borrowers are more likely to issue a specific GSS bond type when they have already issued the same type of non-conventional security. For example, Sustainability issuers are more likely than non Sustainability issuer to issue a sustainability bond (9.8 % as opposed to 0.3 %) rather than a green bond (3.5 % as opposed to 1.7 %). This suggests that specificities at the level of security types, for instance in terms of reporting and transparency requirements, make learning particularly compelling for non-conventional bonds.

**Table 7:** Propensity to issue GSS bonds and experience in the non-conventional bond market. In all panels the dependent variable equals zero (one) in years in which a firm issues no bonds (conventional bonds only); it equals two (three) [four] in years in which a firm issues green bonds (sustainability bonds) [social bonds]. Greenissuer (Sustainabilityissuer) [Socialissuer] is a dummy variable equal to one if the borrower has already issued a green bond (sustainability bond) [social bond] in previous years. Greenissuerwithext.assurance (Sustainabilityissuerwithext.assurance) [Socialissuerwithext.assurance] is a dummy variable equal to one if the borrower has already issued a green bond (sustainability bond) [social bond] with external assurance (i.e., second party opinion, verification, or certification) in previous years. All models include the set of control variables reported in Table 2, and defined in Table A.2, and year times geographic area fixed effects. Standard errors are robust to heteroscedasticity and clustered at the issuer level; t-stats are reported in parentheses and significance levels are reported as follows: ***p<0.01, **p<0.05, *p<0.1.

Outcome	Conv	Green	Sust	Soc
	I	II	III	IV
Panel A				
Green issuer	0.1333	2.8187***	1.6990***	1.3869***
	(0.1043)	(0.1119)	(0.2225)	(0.2633)
Sustainability issuer	−0.1386	1.0998***	3.8509***	2.1954***
	(0.2830)	(0.3864)	(0.3269)	(0.3883)
Social issuer	0.1738	1.3167***	2.5729***	4.1457***
	(0.2310)	(0.3677)	(0.3791)	(0.2826)
Observations	60,967	60,967	60,967	60,967
Pseudo R-squared	0.0554	0.0554	0.0554	0.0554
Macro & firm controls	Yes	Yes	Yes	Yes
Year FE × Geo FE	Yes	Yes	Yes	Yes
Panel B				
Green issuer with ext. assurance	−0.1190	2.041***	0.4789	−0.0072
	(−0.2219)	(0.5874)	(0.3820)	(−0.0261)
Sustainability with ext. assurance	0.2127	0.2835	2.6851***	1.8420**
	(0.2542)	(0.3201)	(0.7054)	(0.8477)
Social issuer with ext. assurance	1.1269	1.1421	1.5658	2.8212***
	(1.0457)	(0.7894)	(1.0125)	(0.5813)
Observations	60,967	60,967	60,967	60,967
Pseudo R-squared	0.0556	0.0556	0.0556	0.0556
Macro & firm controls	Yes	Yes	Yes	Yes
Year FE × Geo FE	Yes	Yes	Yes	Yes

Along the same lines, we then investigate whether the credentials of the previous GSS issues affect the probability of raising additional funds in the same market segment. For that, we consider only previous issues of non-conventional bonds with external assurance, that is those for which the borrower acquired a second party opinion, verification, or certification by the Climate Bond Initiative (CBI). Then, we define a corresponding dummy for each non-conventional bond type issue. The results in Panel B of Table 7 suggest that previous issuance of green, sustainability and social bonds with external assurance significantly increases the probability of issuing the same type of non-conventional bonds. These results confirm the role of costly external assurance as a stronger and more credible signal of sustainability commitment.

## 5 Additional evidence

### 5.1 Amounts issued

So far we have focused on the extensive margin in using the GSS bond market, that is on the probability of issuing non-conventional bonds and conventional fixed income securities. A natural extension is to bring also the intensive margin into the picture. For that, we employ a double hurdle model. This approach models bond issuance as the result of two outcomes. The first outcome refers to the decision to enter the bond market (participation equation) and the second concerns the amount of finance raised (quantity equation), conditional on the first outcome. This empirical setting allows us to uncover the determinants of selection into the bond market as well as the extent of its use, possibly using different sets of variables in the corresponding equations.^22^

Formally, the participation equation is defined as:

(2) $$\begin{array}{ll} xd_{i,m,t} & \;=\left\{ \begin{array}{ll} 1 & \text{if}\;d_{i,m,t}^{*}\;>\;0\\ 0 & \text{otherwise} \end{array}\right. \end{array}$$

(3) $$d_{i,m,t}=\gamma^{\prime }X+v_{i,m,t}$$

where $d_{i,m,t}$ is a latent variable taking the value 1 if firm i participates in the bond market m at time t, and 0 otherwise. Similarly to Section 3.2, X is a vector of domestic and issuer-level explanatory variables, including the same set of controls. We consider year × geographic area fixed effect and cluster the standard errors at issuer level. The quantity equation is defined as:

(4) $$\begin{array}{ll} y_{i,m,t} & \;=\left\{ \begin{array}{ll} 1 & \text{if}\;y_{i,m,t}^{*}\;>\;0\;\text{and}\;d_{i,m,t}^{*}\;>\;0\\ 0 & \text{otherwise} \end{array}\right. \end{array}$$

(5) $$y_{i,m,t}=\gamma^{\prime }X+u_{i,m,t}$$

where $y_{i,m,t}$ denotes a positive level of bonds issued only if the borrower participates in the bond market m at time t and issues a positive amount ($y_{i,m,t}^{*}>0$). We define $y_{i,m,t}$ as the ratio of the amount issued for bond type m over total assets in year t. We estimate separate models for the choice of green, sustainability and social bonds. This implies that, unlike the multinomial logit framework that assumes a simultaneous decision among all the alternative bond types, here the participation equation models a binary decision, i.e., only whether an issuer uses or not each bond type. Still, a double hurdle model provides further insights with the quantity equation, which captures the determinants of issued amounts.

Results are reported in Table A.6 in the Appendix. Model (1) shows the estimates for the green bond issues, model (2) for sustainability bonds, and model (3) for social bonds. For each model specification, odd-(even-)numbered columns in Roman numerals show the results of the participation (quantity) equation. The coefficients in column I are qualitatively similar to the ones obtained from the multinomial logit model (see column IV in Table 3). Higher inflation and lower GDP growth negatively affect the decision to issue green bonds. Additionally, green bond issuance is more likely when issuers are state-owned, larger in size, less leveraged and more profitable. By contrast, conditional on having issued, smaller, non-public and younger issuers raise a larger amount per unit of assets (column II). Macroeconomic variables do not seem to affect green issued amounts.

Looking at the sustainability bond issues in columns III and IV, we observe that both the decision to issue and the amount raised are counter-cyclical. Similarly to the green bond market, we find that smaller and younger issuers are less likely to issue sustainability bonds but, when they do, they raise larger amounts. State-owned and non-listed firms are also more likely to access the sustainable bond segment. Finally, column V suggests that social bonds are more likely to be issued when GDP growth is lower, and by borrowers that are public, larger, more profitable and older. Column VI shows that the amount of finance raised with social bonds decreases when borrowers are larger and listed.

While volumes of GSS debt are still very small compared to conventional bond issues, this analysis provides some indications on the dynamics and the prospects of the sustainable segment. The choice of small issuers to raise relatively large amounts in the face of limited participation is suggestive of possibly high costs to enter the sustainable segment. Being a public issuer is important only for the decision to participate in the GSS bond market but does not affect the amount of sustainability and social bonds, and the effect is even reversed for green bonds. Also other variables that are usually relevant for the access to the debt market, such as firm age and being publicly listed, do not play an important role in the use of GSS securities at the intensive margin. This indicates that issuers do not face severe constraints in terms of amounts, as confirmed by the sustained investor demand for GSS bonds.

Next, we explore in this setting the role of country’s sustainability characteristics and policies by running separate models for green, sustainability and social bonds (see Table A.7 in Appendix), and considering the different variables of interest in each panel. When looking at the coefficients of the participation equation for all three types of non-conventional bonds (columns I, III, and V), the double-hurdle models confirm the findings in Table 4. In addition, results from the quantity equation provide the following insights. In Panel A, ND-GAIN has a positive and significant effect on the amount of funds raised by issuers in the green bond market. In Panel B, Country E-pillar does not affect the amount of green bonds but it has a positive effect on the amount of sustainability bonds. The positive coefficient of the variable Country S-pillar suggests that higher indicators on the policy dimension increase the amount of social bonds. Hence, country indicators related to sustainability seem to be important not only for the access to specific market segments but, in some cases, also for the amount raised. Differently, while the variable Sovereign GSS has a relevant role in access to the GSS market, it does not affect the amount of funds (Panel C). We also test Hypotheses 2A–2C. Results are displayed in Table A.8 in Appendix. In most cases previous results (see Table 5, Table 6, Table 7) on the probability of issuing a certain GSS are confirmed. Some additional patterns can be identified. While issuers with higher ESG scores have a similar participation in the green bond market as other issuers, they raise a higher amount of green funds (Panel A). This last result is in line with Sharfman and Fernando (2008), who find that environmental risk management increases the leverage ratio. The dummy variable Taxonomy also has a positive impact on the amount of green bonds (Panel B). Importantly operating in HtA or transition sectors while increasing the probability of issuing a social bonds reduces the amount of social bonds (Panels C and D). Finally, previous issuances in a specific GSS bond market increase not only the probability of issuing that specific bond type but also its amount (Panel E).^23^ Overall, this analysis corroborates the view that those factors identified as important to access green, sustainability and social bond markets are not necessarily associated with larger amounts of green, sustainability and social bonds. Notice that concerning the amounts of funds raised, the specificities of the project could also be important; hence as these market segments will expand, additional investigations are necessary.

### 5.2 Sustainability-linked bonds

In this section we explore the drivers behind the choice of a different type of non-conventional securities, namely sustainability-linked bonds (SLBs). As explained in Section 3.1, SLBs finance activities that are part of the borrower’s sustainability strategy, but, unlike green, sustainability and social bonds, they are not tied to specific investment projects. Being linked to the sustainability performance of the issuer, their contractual features are different from those of other securities (Barbalau and Zeni, 2022, Berrada et al., 2022, Kölbel and Lambillon, 2022, Fatica, 2025). In Table A.9 in Appendix we add SLBs as a separate outcome of the dependent variable (i.e., B=5 in model (1), if a company has issued an SLB in the year). Column V suggests that, in a similar fashion to sustainability bonds, sustainability-linked issues tend to be negatively correlated with the business cycle and are more likely issued by larger and older borrowers. Overall, SLBs account for a very small fraction of bonds in our sample (153 observations only in the issuer-year panel). Further investigation is warranted to derive more robust conclusion on the specificities of SLBs’ issuers once this instrument has gained traction and a larger number of issues becomes available. Focusing on columns I–IV, we notice that the estimates are remarkably similar to the ones in Table 3, which lends support to our model choice.

## 6 Conclusion

In this paper, we analyze the determinants of green, sustainability and social bond issuance using a large sample of corporate issuers for the years 2014–22. We highlight the importance of countries’ sustainability stance in promoting corporate access to the sustainable bond segment, as well as the role of issuer features and commitment related to sustainability. Borrowers in sectors that are green or can become green, with better environmental and governance scores, and who are experienced and credible as GSS issuers, are more likely to raise funds through non-conventional securities. These results are reassuring against GSS issues being used as sustainability-washing practices. Formally testing for these motives is important to fully uncover the risks and prospects of the non-conventional bond market. However, it requires ad-hoc empirical settings and data on quantifiable sustainability outcomes at the corporate level that are still scant. Therefore, we leave that for future research.

Our analysis complements previous works on corporate funding choices, and is more strictly related to the growing literature that focuses on innovative debt products. In highlighting how a favorable policy stance acts as an incentive for corporate access to the GSS segment, our results have important implications for policymakers and regulators who want to promote funding opportunities of projects with environmental and social benefits. At the same time, our results point to some differences in how the macroeconomic and firm-level fundamentals influence the choice of debt instruments, even within the GSS category. However, with the sustainable debt segment still far from reaching maturity, it is arguably too early to identify structural patterns and a specific profile of sustainable borrowers.

As the sustainable bond segment continues to expand and more standardized frameworks are adopted, other analyses are warranted, particularly to shed light on the different risks and incentives embedded in use-of-proceeds versus contingent securities, such as sustainability-linked bonds. Future research may also include other external financing sources related to sustainable products, such as sustainable loans (on the syndicated loan market see Aleszczyk et al. (2022) and Pinto et al. (2024)). This would have implications for the overall corporate debt structure, particularly for companies that require more resources to transition towards sustainability.

## CRediT authorship contribution statement

**Annette Becker:** Data curation, methodology, and visualization. **Serena Fatica:** Writing – review & editing, writing – original draft, and conceptualization. **Michela Rancan:** Methodology, formal analysis, and conceptualization.

## Disclaimer

The views expressed are purely those of the authors and should not, under any circumstances, be regarded as stating an official position of the European Commission. All remaining errors are our own.

## Figures and Tables

**Table A.1:** List of countries with GSS issuers included in the sample.

Angola	Ghana	Paraguay
Argentina	Greece	Peru
Australia	Guatemala	Philippines
Austria	Honduras	Poland
Bangladesh	Hungary	Portugal
Barbados	Iceland	Puerto Rico
Belarus	India	Qatar
Belgium	Indonesia	Romania
Bermuda	Ireland	Russian Federation
Bolivia	Israel	Saudi Arabia
Botswana	Italy	Serbia
Brazil	Jamaica	Singapore
Bulgaria	Japan	Slovakia
Cambodia	Kazakhstan	Slovenia
Canada	Kenya	South Africa
Chile	Kuwait	South Korea
China	Laos	Spain
Colombia	Latvia	Sri Lanka
Costa Rica	Lithuania	Sweden
Cote d’Ivoire	Luxembourg	Switzerland
Croatia	Malaysia	Thailand
Cyprus	Malta	Togo
Czechia	Mauritius	Trinidad and Tobago
Denmark	Mexico	Tunisia
Dominican Republic	Moldova	Turkey
Ecuador	Monaco	Turks and Caicos Islands
Egypt	Mongolia	Ukraine
El Salvador	Morocco	United Arab Emirates
Estonia	Netherlands	United Kingdom
Faroe Islands	New Zealand	United States
Finland	Nigeria	Uruguay
France	Norway	Uzbekistan
Georgia	Oman	Venezuela
Germany	Panama	Vietnam

**Table A.2:** Variable definitions. This table reports the variable definitions and the data sources.

Variable	Definition	Source
VIX	Stock market volatility index	Bloomberg
Inflation	Consumer price index inflation rate (%)	World Bank
GDP growth	Real growth rate (%) of national GDP	World Bank
Public issuer	Dummy variable set equal to one for the state-owned issuers	DCM, Orbis
Size	Book value of assets (in logs)	Orbis
Leverage	Total liabilities scaled by the book value of total assets	Orbis
ROA	Profits scaled by the book value of total assets	Orbis
Age	Difference between the current year and the founding year of the firm	Orbis
Listed	Dummy variable equal to one if an issuer is listed	Orbis
ND-GAIN index	The Notre Dame Global Adaptation Initiative’s index measuring the climate readiness of a country	gain.nd.edu
Country E-pillar	1st component of the PCA on the country environmental indicators	World Bank
Country S-pillar	1st component of the PCA on the country social indicators	World Bank
Country G-pillar	1st component of the PCA on the country governance indicators	World Bank
Sovereign GSS	Dummy variable equal to one for issuers located in countries with previous sovereign GSS issues	DCM
ESG	Borrower ESG score	Refinitiv
E score	Borrower environmental score (E component of the ESG score)	Refinitiv
S score	Borrower social score (S component of the ESG score)	Refinitiv
G score	Borrower governance score (G component of the ESG score)	Refinitiv
Taxonomy	Dummy variable equal to one for issuers in taxonomy-eligible sectors	DCM, Orbis
Hard-to-abate	Dummy equal to one for issuers in hard-to-abate sectors	DCM, Orbis
Transition	Dummy equal to one for issuers in transition sectors	DCM, Orbis
Green issuer	Dummy variable equal to one for borrowers with previous green bond issues	DCM
Sustainability issuer	Dummy variable equal to one for borrowers with previous sustainability bond issues	DCM
Social issuer	Dummy variable equal to one for borrowers with previous social bond issues	DCM
Green issuer with ext. assurance	Dummy variable equal to one for borrowers with previous green bond issues with external assurance	DCM
Sustainability issuer with ext. assurance	Dummy variable equal to one for borrowers with previous sustainability bond issues with external assurance	DCM
Social issuer with ext. assurance	Dummy variable equal to one for borrowers with previous social bond issues with external assurance	DCM

**Table A.3:** Summary statistics: independent variables. This table provides descriptive statistics for the variables used in the analysis.

Variable	N	Mean	St. dev.	P.le 50	P.le 5	P.le 95
ND-GAIN	60911	63.64	8.32	67.78	48.14	72.83
Country E-pillar	31209	−1.05	1.47	−1.74	−2.67	1.81
Country S-pillar	31209	2.51	1.59	3.16	−0.88	3.80
Country G-pillar	31209	0.82	1.94	1.71	−2.13	3.40
Sovereign GSS	60967	0.10	0.30	0.00	0.00	1.00
ESG score	16302	50.68	29.25	58.86	0.42	86.97
E score	16302	49.45	33.18	57.14	0.17	91.78
S score	16302	51.27	31.13	58.60	0.41	92.42
G score	16302	50.42	29.95	56.71	0.46	90.16
Taxonomy sector	60499	0.14	0.35	0.00	0.00	1.00
Hard-to-abate sector	60499	0.04	0.20	0.00	0.00	0.00
Transition sector	60499	0.04	0.21	0.00	0.00	0.00
Green issuer	60967	0.02	0.15	0.00	0.00	0.00
Sustainability issuer	60967	0.00	0.06	0.00	0.00	0.00
Social issuer	60967	0.00	0.06	0.00	0.00	0.00
Green issuer with ext. assurance	60967	0.00	0.05	0.00	0.00	0.00
Sustainability issuer with ext. assurance	60967	0.00	0.02	0.00	0.00	0.00
Social issuer with ext. assurance	60967	0.00	0.02	0.00	0.00	0.00

**Table A.4:** NACE sectors of taxonomy-eligible activities.

Taxonomy sectors	
A1.1 - Growing of non-perennial crops	E37.0 - Sewerage
A1.2 - Growing of perennial crops	E38.1.1 - Collection of non-hazardous waste
A1.4 - Animal production	E38.2.1 - Treatment and disposal of non-hazardous waste
C20.1.1 - Manufacture of industrial gases	E38.3.2 - Recovery of sorted materials
C20.1.3 - Manufacture of other inorganic basic chemicals	E39.0 - Remediation activities and other waste management services
C20.1.4 - Manufacture of other organic basic chemicals	F41.2 - Construction of residential and non-residential buildings
C20.1.5 - Manufacture of fertilisers and nitrogen compounds	F42.1 - Construction of roads and railways
C20.1.6 - Manufacture of plastics in primary forms	F42.9.1 - Construction of water projects
C23.5.1 - Manufacture of cement	H49.1.0 - Passenger rail transport, interurban
C24.4.2 - Aluminium production	H49.2.0 - Freight rail transport
C24.5.1 - Casting of iron	H49.3.1 - Urban and suburban passenger land transport
C24.5.2 - Casting of steel	H49.3.9 - Other passenger land transport n.e.c.
D35.1.1 - Production of electricity	H49.4.1 - Freight transport by road
D35.1.2 - Transmission of electricity	H49.5.0 - Transport via pipeline
D35.2.1 - Manufacture of gas	H50.3.0 - Inland passenger water transport
D35.3 - Steam and air conditioning supply	H50.4.0 - Inland freight water transport
E36.0 - Water collection, treatment and supply	J63.1.1 - Data processing, hosting and related activities

**Table A.5:** NACE sectors of hard-to-abate and transition activities.

HTA sectors	
B7.1.0 - Mining of iron ores	C23.4 - Manufacture of other porcelain and ceramic products
B7.2 - Mining of non-ferrous metal ores	C23.9.9 - Manufacture of other non-metallic mineral products n.e.c.
B8.1 - Quarrying of stone, sand and clay	C24.1.0 - Manufacture of basic iron and steel and of ferro-alloys
B8.9 - Mining and quarrying n.e.c.	C26.1 - Manufacture of electronic components and boards
C17.2.1 - Manufacture of corrugated paper and paperboard	C32.5.0 - Manufacture of medical and dental instruments and supplies
C17.2.2 - Manufacture of household and sanitary goods and of toilet requisites	H49.1.0 - Passenger rail transport, interurban
C19.2.0 - Manufacture of refined petroleum products	H49.4 - Freight transport by road and removal services
C20.5 - Manufacture of other chemical products	H49.5.0 - Transport via pipeline
C23.1.0 - Manufacture of glass and glass products	H50.1.0 - Sea and coastal passenger water transport
C23.3 - Manufacture of clay building materials	H51.2 - Freight air transport and space transport

Transition sectors	
C20.1.3 - Manufacture of other inorganic basic chemicals	C24.5.2 - Casting of steel
C20.1.4 - Manufacture of other organic basic chemicals	C24.5.3 - Casting of light metals
C20.1.5 - Manufacture of fertilisers and nitrogen compounds	H49.1.0 - Passenger rail transport, interurban
C20.1.6 - Manufacture of plastics in primary forms	H49.2.0 - Freight rail transport
C23.5.1 - Manufacture of cement	H49.3.1 - Urban and suburban passenger land transport
C24.1.0 - Manufacture of basic iron and steel and of ferro-alloys	H49.4.1 - Freight transport by road
C24.2.0 - Manufacture of tubes, pipes, hollow profiles and related fittings, of steel	H50.3.0 - Inland passenger water transport
C24.3 - Manufacture of other products of first processing of steel	H50.4.0 - Inland freight water transport
C24.4.2 - Aluminum production	J63.1 - Data processing, hosting and related activities
C24.5.1 - Casting of iron	

**Table A.6:** Double hurdle model. This table reports the results of a double hurdle model for issuer activity in the green (model (1)), sustainability (model (2)) and social (model (3)) bond market. Odd-numbered columns in roman numerals report the results for the participation equation, which models the probability of issuing the corresponding non-conventional bond type in a given year. Even-numbered columns in roman numerals report the results for the quantity equation, which models the volumes issued conditional on accessing the corresponding non-conventional bond type in a given year. Standard errors, robust to heteroscedasticity and clustered at the issuer level, are reported in parentheses. Significance levels are reported as follows: ***p<0.01, **p<0.05, *p<0.1.

	(1) Green		(2) Sust		(3) Soc	
	Participation	Quantity	Participation	Quantity	Participation	Quantity
	Eq. I	Eq. II	Eq. III	Eq. IV	Eq. V	Eq. VI
VIX	0.0010	−0.0155	0.0004	−0.0390	0.0832*	0.0572
	(0.008)	(0.019)	(0.013)	(0.066)	(0.046)	(0.090)
Inflation	−0.0219***	0.0115	−0.0272**	−0.0302	−0.0190	0.0281
	(0.005)	(0.014)	(0.013)	(0.023)	(0.012)	(0.021)
GDP growth	0.0197***	−0.0098	−0.0720***	−0.0749**	−0.0469***	0.0273
	(0.007)	(0.013)	(0.014)	(0.033)	(0.012)	(0.036)
Public issuer	0.3087***	−0.2049**	0.5860***	0.3318	0.4926***	−0.0996
	(0.055)	(0.093)	(0.081)	(0.275)	(0.088)	(0.219)
Size	0.0779***	−0.4247***	0.0622***	−0.8540***	0.0770***	−0.6799***
	(0.009)	(0.024)	(0.016)	(0.074)	(0.021)	(0.048)
Leverage	−0.2049**	−0.5262**	−0.2530	1.0161	−0.2585	−0.5796
	(0.087)	(0.247)	(0.218)	(0.642)	(0.188)	(0.448)
ROA	1.2024***	0.2700	0.5892	3.4085	1.5094*	−0.7342
	(0.300)	(0.798)	(0.688)	(3.536)	(0.791)	(2.488)
Age	−0.0000	−0.0021*	0.0027***	−0.0043**	0.0015**	0.0016
	(0.001)	(0.001)	(0.001)	(0.002)	(0.001)	(0.002)
Listed	−0.0743*	−0.1177	−0.2354***	−0.1826	0.0266	−0.6420***
	(0.044)	(0.074)	(0.064)	(0.217)	(0.065)	(0.207)
Constant	−3.6948***	3.4885***	−3.9802***	8.0398***	−5.6661***	5.9208***
	(0.185)	(0.502)	(0.315)	(1.760)	(0.815)	(1.897)
Observations	60,967	60,967	60,967	60,967	60,967	60,967
Year FE × Geo FE	Yes	Yes	Yes	Yes	Yes	Yes

**Table A.7:** Double hurdle model for GSS bonds and country sustainability. This table reports the results of a double hurdle model for issuer activity in the green (model (1)), sustainability (model (2)) and social (model (3)) bond market. Odd-numbered columns in roman numerals report the results for the participation equation, which models the probability of issuing the corresponding non-conventional bond type in a given year. Even-numbered columns in roman numerals report the results for the quantity equation, which models the volumes issued conditional on accessing the corresponding non-conventional bond type in a given year. In panel A, ND−GAIN is a measure of a country’s preparedness for climate change. In panel B, CountryE−pillar is the 1st component of the PCA on the country environmental indicators; CountryS−pillar is the 1st component of the PCA on the country social indicators; CountryG−pillar is the 1st component of the PCA on the country governance indicators. In panel C, SovereignGSS is a dummy variable equal to one if sovereign GSS bonds have been issued in the jurisdiction where the borrower is incorporated„ and zero otherwise. All models include the set of control variables reported in Table 2 and year × geographic area fixed effects. Standard errors, robust to heteroscedasticity and clustered at the issuer level, are reported in parentheses. Significance levels are reported as follows: ***p<0.01, **p<0.05, *p<0.1.

	(1) Green		(2) Sust		(3) Soc	
	Participation	Quantity	Participation	Quantity	Participation	Quantity
	Eq. I	Eq. II	Eq. III	Eq. IV	Eq. V	Eq. VI
Panel A						
ND-GAIN	0.0136***	0.0313***	0.0048	−0.0007	0.0139***	−0.0216
	(0.003)	(0.010)	(0.007)	(0.014)	(0.005)	(0.017)
Observations	60,967	60,967	60,967	60,967	60,967	60,967
Macro & firm controls	Yes	Yes	Yes	Yes	Yes	Yes
Year FE × Geo FE	Yes	Yes	Yes	Yes	Yes	Yes
Panel B						
Country E-pillar	0.2484**	−0.6104	−0.0956	1.0415***	−0.4654	1.3786
	(0.106)	(0.598)	(0.140)	(0.314)	(0.294)	(0.990)
Country S-pillar	0.0689	0.1930	−0.0559	−0.4614	0.2596**	0.3917***
	(0.059)	(0.125)	(0.086)	(0.324)	(0.104)	(0.142)
Country G-pillar	0.3596***	−0.9072	0.0156	−1.0256	−0.2951	0.4130
	(0.079)	(0.888)	(0.129)	(0.846)	(0.215)	(0.320)
Observations	60,967	60,967	60,967	60,967	60,967	60,967
Macro & firm controls	Yes	Yes	Yes	Yes	Yes	Yes
Year FE × Geo FE	Yes	Yes	Yes	Yes	Yes	Yes
Panel C						
Sovereign GSS	0.1370***	−0.0839	0.0594	0.1889	0.1899**	−0.3235
	(0.050)	(0.114)	(0.079)	(0.205)	(0.080)	(0.289)
Observations	60,967	60,967	60,967	60,967	60,967	60,967
Macro & firm controls	Yes	Yes	Yes	Yes	Yes	Yes
Year FE × Geo FE	Yes	Yes	Yes	Yes	Yes	Yes

**Table A.8:** Double hurdle model for GSS bonds and issuer sustainability profile. This table reports the results of a double hurdle model for issuer activity in the green (model (1)), sustainability (model (2)) and social (model (3)) bond market. Odd-numbered columns in roman numerals report the results for the participation equation, which models the probability of issuing the corresponding non-conventional bond type in a given year. Even-numbered columns in roman numerals report the results for the quantity equation, which models the volumes issued conditional on accessing the corresponding non-conventional bond type in a given year. In panel A, ESG is the issuer ESG score as defined by Refinitiv. In panel B, Taxonomy is a dummy variable equal to one if the issuer’s sector is among the eligible sectors according to the EU taxonomy of sustainable activities, and zero otherwise. In panel C, Hard−to−abate is a dummy variable equal to one if the issuer’s sector is classified as hard-to-abate. In panel D, Transition is a dummy variable equal to one if the issuer’s sector is included in the list of transition sectors. In panel E Greenissuer (Sustainabilityissuer) [Socialissuer] is a dummy variable equal to one f the borrower has already issued a green bond (sustainability bond) [social bond] in previous years. All models include the set of control variables reported in Table 2 and year × geographic area fixed effects. Standard errors, robust to heteroscedasticity and clustered at the issuer level, are reported in parentheses. Significance levels are reported as follows: ***p<0.01, **p<0.05, *p<0.1.

	(1) Green		(2) Sust		(3) Soc	
	Participation	Quantity	Participation	Quantity	Participation	Quantity
	Eq. I	Eq. II	Eq. III	Eq. IV	Eq. V	Eq. VI
Panel A						
ESG	0.0026	0.0130***	0.0015	0.0012	0.00106	−0.0000
	(0.002)	(0.003)	(0.002)	(0.008)	(0.002)	(0.003)
Observations	16,302	16,302	16,302	16,302	16,302	16,302
Macro & firm controls	Yes	Yes	Yes	Yes	Yes	Yes
Year FE × Geo FE	Yes	Yes	Yes	Yes	Yes	Yes
Panel B						
Taxonomy	0.3424***	0.3553***	−0.1849**	−0.2846	0.1174	−0.2721
	(0.046)	(0.084)	(0.092)	(0.276)	(0.081)	(0.227)
Observations	60,967	60,967	60,967	60,967	60,967	60,967
Macro & firm controls	Yes	Yes	Yes	Yes	Yes	Yes
Year FE × Geo FE	Yes	Yes	Yes	Yes	Yes	Yes
Panel C						
Hard-to-abate	−0.0523	−0.0649	−0.2434	0.5427	0.2147*	−0.8589***
	(0.081)	(0.201)	(0.178)	(0.373)	(0.121)	(0.252)
Observations	60,967	60,967	60,967	60,967	60,967	60,967
Macro & firm controls	Yes	Yes	Yes	Yes	Yes	Yes
Year FE × Geo FE	Yes	Yes	Yes	Yes	Yes	Yes
Panel D						
Transition	0.1429***	−0.0151	−0.1667	−0.5549	0.2075*	−0.5403*
	(0.028)	(0.189)	(0.141)	(0.397)	(0.119)	(0.320)
Observations	60,967	60,967	60,967	60,967	60,967	60,967
Macro & firm controls	Yes	Yes	Yes	Yes	Yes	Yes
Year FE × Geo FE	Yes	Yes	Yes	Yes	Yes	Yes
Panel E						
Green issuer	1.3715***	0.1430**	0.5920***	−0.0331	0.4360***	0.5875*
	(0.055)	(0.069)	(0.083)	(0.229)	(0.097)	(0.315)
Sustainability issuer	0.8748***	0.3134	2.0285***	0.0837***	0.8311***	−0.3896
	(0.173)	(0.221)	(0.142)	(0.196)	(0.152)	(0.432)
Social issuer	0.7093***	−0.0353	1.3250***	0.0071	1.9812***	0.5677***
	(0.149)	(0.355)	(0.135)	(0.225)	(0.111)	(0.178)
Observations	60,967	60,967	60,967	60,967	60,967	60,967
Macro & firm controls	Yes	Yes	Yes	Yes	Yes	Yes
Year FE × Geo FE	Yes	Yes	Yes	Yes	Yes	Yes

**Table A.9:** Sustainability-linked bonds. This table reports the results of a multinominal logit model of issuer activity in the bond market, with sustainability-linked bonds included as a separate category. In all panels the dependent variable equals zero (one) in years in which a firm issues no bonds (conventional bonds only); it equals two (three) [four] {five} in years in which a firm issues green bonds (sustainability bonds) [social bonds] {sustainability-linked bonds}. Control variables are defined in Table A.2. All models include year × geographic area fixed effects. Standard errors are robust to heteroscedasticity and clustered at the issuer level; t-stats are reported in parentheses and significance levels are reported as follows: ***p<0.01, **p<0.05, *p<0.1.

Outcome	Conv	Green	Sust	Soc	SLB
	I	II	III	IV	V
VIX	−0.0285***	−0.0077	−0.0073	0.4727	−0.1360***
	(0.0043)	(0.0217)	(0.0465)	(0.3981)	(0.0431)
Inflation	−0.0126***	−0.0593***	−0.1733***	−0.0541*	−0.0332
	(0.0033)	(0.0134)	(0.0300)	(0.0291)	(0.0277)
GDP growth	−0.0147**	0.0405**	−0.1859***	−0.1388***	−0.1086***
	(0.0064)	(0.0168)	(0.0635)	(0.0314)	(0.0312)
Public issuer	0.1884***	0.7325***	2.7192***	1.4271***	−0.5919*
	(0.0449)	(0.1289)	(0.2842)	(0.2517)	(0.3204)
Size	0.0110	0.1700***	0.2244***	0.2178***	0.0954**
	(0.0069)	(0.0209)	(0.0794)	(0.0621)	(0.0433)
Leverage	0.0980	−0.5499**	−0.7463	−0.9859*	−0.3766
	(0.0679)	(0.2170)	(0.9394)	(0.5325)	(0.4779)
ROA	2.2317***	3.2027***	2.9005	3.5327	1.5227
	(0.2271)	(0.7268)	(4.9554)	(2.2133)	(1.4522)
Age	0.0012***	−0.0006	0.0047**	0.0040**	0.0068***
	(0.0004)	(0.0013)	(0.0021)	(0.0020)	(0.0016)
Listed	0.1008***	−0.0884	−0.6261**	0.1076	−0.3870**
	(0.0311)	(0.1060)	(0.2550)	(0.1819)	(0.1938)
Observations	60,967	60,967	60,967	60,967	60,967
of which Y=1	16,706	16,706	16,706	16,706	16,706
of which Y=2	1,059	1,059	1,059	1,059	1,059
of which Y=3	153	153	153	153	153
of which Y=4	236	236	236	236	236
of which Y=5	153	153	153	153	153
Pseudo R-squared	0.0391	0.0391	0.0391	0.0391	0.0391
Year × Geo FE	Yes	Yes	Yes	Yes	Yes

## Data Availability

The authors do not have permission to share data.

## Footnotes

1 We refer interchangeably to GSS, sustainable and non-conventional bonds.

2 Energy efficiency projects include power sector investments, such as electricity transmission and smart grids, and technologies to reduce carbon intensity, such as carbon capture, usage and storage.

3 This corroborates evidence in Fatica and Panzica (2021) documenting that, in a large international sample of corporate green bonds, roughly 80 % of UoP refer, fully or partly, to climate change mitigation projects.

4 ICMA guidelines: Green, sustainability and social bonds principles.

5 EC: Strategy for financing the transition to a sustainable economy, 2021.

6 EC timeline: The European green bond standard.

7 Tax-based incentive schemes in place for US green issuance included the so-called tax credit bonds, direct subsidy bonds and tax exemptions. Tax credit bonds provide bond investors with tax credits instead of interest payments, so issuers do not have to pay interest on their green bond issuances. With direct subsidy bonds issuers receive cash rebates from the government to subsidize their net interest payments, while tax-exempt bonds exempt investors from federal income taxes on interest from the green bonds they hold.

8 EUR-Lex: Summary of EU legislation on the European green bond standard

9 Eurosif (2021) reports that exclusion strategy is the most popular one followed by engagement and voting, ESG integration, norms-based screening and best-in-class.

10 Torricelli and Pellati (2023) document a positive premium for social bonds in the secondary market.

11 We consider the KPIs, particularly when only environmental indicators were included, as well as other information about the bond. The issue report often provides details on company alignment with United Nations Sustainable Development Goals and other international initiatives. By their nature, the UoP of SLBs usually refers to the company’s sustainability framework or general corporate purposes, without reference to specific investment projects with environmental, social and broader sustainability goals. In Section 5.2 we provide additional evidence considering SLBs separately.

12 See Rancan et al. (2023) for some empirical evidence on the access to the bond market of European banks and Bedendo et al. (2023) more specifically to the green bond market.

13 Table A.1 in the Appendix displays the complete list of countries covered in our sample. We consider the jurisdiction where the issuer’s headquarter is incorporated.

14 As a preliminary step, we also estimate a more compact version of model (1) with only three alternatives, namely no bonds, conventional bonds or any of the GSS securities. See Table 3, model (1).

15 This is a standard approach in this literature (see, e.g., Erel et al., 2012).

16 A key assumption of the multinomial logit model is the independence from irrelevant alternatives (IIA). We formally check whether IIA holds by running a Hausman test for the equality of parameters (Hausman and McFadden, 1984). We find that the IIA holds confirming that the multinomial logit model is valid and unbiased.

17 Summary statistics of this variable and the other variables used in the empirical analysis are reported in Table A.3.

18 After merging these data with our sample, we are left with 16,302 observations for which the ESG score is available.

19 Focusing on the sector allows us to maximize the cross-sectional coverage of the analysis, since firm-level sustainability reporting is still in its infancy, and applies to a limited number of large listed firms.

20 The Taxonomy (EC: EU taxonomy for sustainable activities), the cornerstone of the EU Sustainable Finance Strategy, is structured around six environmental objectives: climate change mitigation; climate change adaptation; sustainable use and protection of water and marine resources; transition to a circular economy; pollution prevention and control; protection and restoration of biodiversity and ecosystems. In an effort to promote international coordination in climate and sustainability action, it serves as a blueprint for the development of similar taxonomies in other jurisdictions across the globe.

21 The detailed list of HtA sector is provided in Table A.5 in Appendix.

22 For comparison, in a Tobit model the participation and the quantity outcomes are assumed to have the same set of determinants.

23 Similar results are obtained when using the variables related to external assurance.

## References

[bib0005] Akerlof G. (1970). The market for “lemons”: quality uncertainty and the market mechanism. Q. J. Econ..

[bib0010] Aleszczyk A.A., Loumioti M., Serafeim G. (2022). The issuance and design of sustainability-linked loans.

[bib0015] Amiraslani H., Lins K.V., Servaes H., Tamayo A. (2023). Trust, social capital, and the bond market benefits of esg performance. Rev. Account. Stud..

[bib0020] Apergis N., Poufinas T., Antonopoulos A. (2022). Esg scores and cost of debt. Energy Econ..

[bib0025] Asimakopoulos P., Asimakopoulos S., Li X. (2023). The role of environmental, social, and governance rating on corporate debt structure. J. Corp. Financ..

[bib0030] Atta-Darkua V., Glossner S., Krueger P., Matos P. (2022). Decarbonizing institutional investor portfolios.

[bib0035] Badoer D.C., James C.M. (2016). The determinants of long-term corporate debt issuances. J. Finance.

[bib0040] Baker M., Bergstresser D., Serafeim G., Wurgler J. (2022). The pricing and ownership of us green bonds. Annu. Rev. Financ. Econ..

[bib0045] Barbalau A., Zeni F. (2022). The optimal design of green securities.

[bib0050] Bartram S.M., Hou K., Kim S. (2022). Real effects of climate policy: financial constraints and spillovers. J. Financ. Econ..

[bib0055] Bedendo M., Nocera G., Siming L. (2023). Greening the financial sector: evidence from bank green bonds. J. Bus. Ethics.

[bib0060] Berg F., Koelbel J.F., Rigobon R. (2022). Aggregate confusion: the divergence of esg ratings. Rev. Financ..

[bib0065] Berrada T., Engelhardt L., Gibson R., Krueger P. (2022). The economics of sustainability linked bonds. Swiss Finance Institute Research Paper 22-26.

[bib0070] Bolton P., Freixas X. (2000). Equity, bonds, and bank debt: capital structure and financial market equilibrium under asymmetric information. J. Polit. Econ..

[bib0075] Bolton P., Kacperczyk M. (2023). Global pricing of carbon-transition risk. J. Finance.

[bib0080] Boot A.W., Thakor A.V. (2000). Can relationship banking survive competition?. J. Finance.

[bib0085] Capelle-Blancard G., Crifo P., Diaye M.-A., Oueghlissi R., Scholtens B. (2019). Sovereign bond yield spreads and sustainability: an empirical analysis of oecd countries. J. Bank. Financ..

[bib0090] Chen H., Maslar D.A., Serfling M. (2020). Asset redeployability and the choice between bank debt and public debt. J. Corp. Financ..

[bib0095] Cheng G., Ehlers T., Packer F., Xiao Y. (2024). Sovereign green bonds: a catalyst for sustainable debt market development?. BIS Working Paper No 1198.

[bib0100] Cicchiello A.F., Cotugno M., Monferrà S., Perdichizzi S. (2022). Which are the factors influencing green bonds issuance? Evidence from the European bonds market. Financ. Res. Lett..

[bib0105] Daubanes J.X., Mitali S.F., Rochet J.-C. (2021). Why do firms issue green bonds?. Swiss Finance Institute Research Paper 21-97.

[bib0110] Delis M.D., Greiff K.D., Iosifidi M., Ongena S. (2024). Being stranded with fossil fuel reserves? Climate policy risk and the pricing of bank loans. Financ. Mark. Inst. Instrum..

[bib0115] Denis D.J., Mihov V.T. (2003). The choice among bank debt, non-bank private debt, and public debt: evidence from new corporate borrowings. J. Financ. Econ..

[bib0120] Diamond D.W. (1991). Monitoring and reputation: the choice between bank loans and directly placed debt. J. Polit. Econ..

[bib0125] Dong H., Zhang L., Zheng H. (2024). Green bonds: Fueling green innovation or just a fad?. Energy Econ..

[bib0130] Dutordoir M., Li S., Neto J.Q.F. (2024). Issuer motivations for corporate green bond offerings. Br. J. Manag..

[bib0135] ElBannan M.A., Löffler G. (2024). How effectively do green bonds help the environment?. J. Bank. Financ..

[bib0140] Erel I., Julio B., Kim W., Weisbach M.S. (2012). Macroeconomic conditions and capital raising. Rev. Financ. Stud..

[bib0145] Eurosif (2021). Eurosif Report 2021–Fostering Investor Impact Placing It at the Heart of Sustainable Finance.

[bib0150] Fabozzi F.J., Ma K., Oliphant B.J. (2008). Sin stock returns. J. Portf. Manag..

[bib0155] Fard A., Javadi S., Kim I. (2020). Environmental regulation and the cost of bank loans: international evidence. J. Financ. Stab..

[bib0160] Fatica S., Caporale G.M. (2025).

[bib0165] Fatica S., Panzica R. (2021). Green bonds as a tool against climate change?. Business strategy and the environment.

[bib0170] Fatica S., Panzica R., Rancan M. (2021). The pricing of green bonds: are financial institutions special?. J. Financ. Stab..

[bib0175] Ferrell A., Liang H., Renneboog L. (2016). Socially responsible firms. J. Financ. Econ..

[bib0180] Fitch (2023). ESG Ratings Insights: Use of Proceeds in Instrument Ratings.

[bib0185] Flammer C. (2021). Corporate green bonds. J. Financ. Econ..

[bib0190] Flannery M.J., Hong C.Y., Wang B. (2023). The effect of government reference bonds on corporate borrowing costs: evidence from a natural experiment. Management Science.

[bib0195] Frank M.Z., Goyal V.K. (2009). Capital structure decisions: which factors are reliably important?. Financ. Manage..

[bib0200] Ginglinger E., Moreau Q. (2023). Climate risk and capital structure. Manage. Sci..

[bib0205] Gomes A., Phillips G. (2012). Why do public firms issue private and public securities?. J. Financ. Intermed..

[bib0210] Guesmi K., Makrychoriti P., Pyrgiotakis E.G. (2025). Climate change exposure and green bonds issuance. J. Int. Money Finance.

[bib0215] Hausman J., McFadden D. (1984). Specification tests for the multinomial logit model. Econom.: J. Econom. Soc..

[bib0220] Hoepner A., Oikonomou I., Scholtens B., Schröder M. (2016). The effects of corporate and country sustainability characteristics on the cost of debt: an international investigation. J. Bus. Finance Account..

[bib0225] Hoepner A.G., Schopohl L. (2020). State pension funds and corporate social responsibility: do beneficiaries’ political values influence funds’ investment decisions?. J. Bus. Ethics.

[bib0230] Hong H., Kacperczyk M. (2009). The price of sin: the effects of social norms on markets. J. Financ. Econ..

[bib0235] Houston J.F., Shan H. (2022). Corporate esg profiles and banking relationships. Rev. Financ. Stud..

[bib0240] Huang Y., Xu J. (2024).

[bib0245] Ivanov I.T., Kruttli M.S., Watugala S.W. (2024). Banking on carbon: corporate lending and cap-and-trade policy. Rev. Financ. Stud..

[bib0250] Jung K., Kim Y.-C., Stulz R. (1996). Timing, investment opportunities, managerial discretion, and the security issue decision. J. Financ. Econ..

[bib0255] Kölbel J.F., Lambillon A.-P. (2022). Who pays for sustainability? An analysis of sustainability-linked bonds. Swiss Finance Institute Research Paper 23-27..

[bib0260] Li D., Magud N.E., Werner A. (2023). The long-run impact of sovereign yields on corporate yields in emerging markets. J. Int. Money Financ..

[bib0265] Liang H., Renneboog L. (2017). On the foundations of corporate social responsibility. J. Financ..

[bib0270] Lin B., Su T. (2022). Green bond vs conventional bond: outline the rationale behind issuance choices in China. Int. Rev. Financ. Anal..

[bib0275] Lins K.V., Servaes H., Tamayo A. (2017). Social capital, trust, and firm performance: the value of corporate social responsibility during the financial crisis. J. Financ..

[bib0280] Liu H., Li Y., Xiao Y., Xiong X., Zhang W. (2024). Social responsibility and corporate borrowing. Eur. J. Financ..

[bib0285] Morellec E., Valta P., Zhdanov A. (2015). Financing investment: the choice between bonds and bank loans. Manag. Sci..

[bib0290] Nguyen J.H., Phan H.V. (2020). Carbon risk and corporate capital structure. J. Corp. Financ..

[bib0295] Pinto J., Alves P., Gonçalo J. (2024). The pricing of sustainable syndicated loans.

[bib0300] Rancan M., Cariboni J., Keasey K., Vallascas F. (2023). Bond issuance and the funding choices of European banks: the consequences of public debt. J. Empir. Finance.

[bib0305] Riley J.G. (1979). Informational equilibrium. Econometrica.

[bib0310] Rossi M., Sansone D., Van Soest A., Torricelli C. (2019). Household preferences for socially responsible investments. J. Bank. Financ..

[bib0315] Sangiorgi I., Schopohl L. (2023). Explaining green bond issuance using survey evidence: beyond the greenium. Br. Account. Rev..

[bib0320] Seltzer, L.H., Starks, L., Zhu, Q., 2022. Climate Regulatory Risk and Corporate Bonds. Tech. Rep. National Bureau of Economic Research.

[bib0325] Sharfman M.P., Fernando C.S. (2008). Environmental risk management and the cost of capital. Strateg. Manag. J..

[bib0330] Shiller R.J. (1994).

[bib0335] Tang D.Y., Zhang Y. (2020). Do shareholders benefit from green bonds?. J. Corp. Financ..

[bib0340] Torricelli C., Pellati E. (2023). Social bonds and the “social premium”. J. Econ. Finance.

[bib0345] Zerbib O.D. (2019). The effect of pro-environmental preferences on bond prices: evidence from green bonds. J. Bank. Financ..

